# A hybrid optimization approach for accelerated multimodal medical image fusion

**DOI:** 10.1371/journal.pone.0324973

**Published:** 2025-07-10

**Authors:** Chisom Ezinne Ogbuanya, Adaora Obayi, Souad Larabi-Marie-Sainte, Amal O. Saad, Lamia Berriche

**Affiliations:** 1 Department of Electronic and Computer Engineering, University of Nigeria, Nsukka, Nigeria; 2 Department of Computer Science, University of Nigeria, Nsukka, Nigeria; 3 Computer Science Department, College of Computer and Information Sciences, Prince Sultan University, Riyadh, Saudi Arabia; 4 Programming Department, Computer Technology Tripoli (Tripoli – Libya), Libya; 5 Computer Science Department, College of Computer and Information Sciences, Prince Sultan University, Riyadh, Saudi Arabia; Northeastern University, CHINA

## Abstract

Multimodal Medical Image Fusion is a key evolution in medical imaging. It contributes to improving diagnosis, providing better treatment, and reducing risk. Multimodal medical image fusion is a multi-objective due to the need of balancing factors like the weights of the fusion rules and the speed of the fusion process. While multi-objective particle swarm optimization has already been applied to solve this problem, it suffers from premature. It has been shown that the Darwinian Particle Swarm Optimization performs better than the classical Particle Swarm optimization by escaping the local optima. Therefore, this paper proposes a new approach based on the combination of variable-order fractional-order with multi-objective Darwinian Particle Swarm Optimization. Variable-order fractional-order improves the convergence rate of multi-objective Darwinian Particle Swarm Optimization by adjusting the particle velocity and position dynamically. Moreover, the new approach uses the gradient compass in the spatial domain to generate detailed images, further enhancing fusion quality. The proposed method is used to optimize both the fusion process weights and processing time. Experiments using the fusion of computed tomography along with magnetic resonance imaging show that the proposed technique outperforms existing techniques. Both the Inverted Generational Distance (IGD) and the Hyper-Volume (HV) metrics of the proposed multi-objective problem solution surpass the state-of-the-art showing the optimality of the provided solution. Additionally, the proposed solution image visual demonstrated high visual quality, efficient edge preservation, and the absence of noisy artefacts. Furthermore, our proposed fusion approach showed its suitability for real-time application, with a processing time not exceeding 0.085 seconds, outperforming other methods.

## 1. Introduction

Medical image fusion consists of a combination of various imaging modalities to generate a single thorough view of a patient’s disease. This process improves diagnostic correctness, enhances treatment planning, and offers better conception of complicated structures. It also helps in detecting abnormalities, resulting in better clinical decisions. Medical image modalities are classified under two types: anatomical or functional. Anatomical imaging modalities, such as X-ray, computed tomography (CT), and magnetic resonance imaging (MRI) focus on the structures of the body [1,2].

An efficient multimodal medical image fusion (MMIF) technique can integrate complementary details from various medical images obtained from one or more imaging modalities to improve visual quality by retaining precise features from tissues or organs for analysis by a physician or for machine recognition [3,4]. For example, Magnetic resonance imaging (MRI) provides details of superior soft-tissue variations, while CT images give insights about thick structures such as bones and implants. Hence, combining MRI and CT images generates comprehensive details for clinical diagnosis and treatment planning. Additionally, the fusion of MRI and CT images, when combined with surgical navigation, assists surgeons in accurately preparing a preoperative plan, potentially improving the surgery outcomes [5,6]. Therefore, multimodal medical image fusion provides relevant technology for the integration of several types of medical imaging information in different clinical applications. CT and MRI images have already been integrated to aid in surgeries for skull base tumors [7]. Additionally, the fusion of CT and MRI images was carried out in [7] and [8] to validate the effectiveness of the novel pulse-coupled neural network (PCNN)-based image integration methods. However, obtaining high-quality fused images results in a short amount of time, especially for processes such as real-time image-guided surgery, is still an unsolved problem [9,10]. This highlights the need to maximize image quality while minimizing fusion time. MMIF can be considered a multi-objective problem that requires a multi-objective algorithm to optimize the fusion process. Recently, many multi-objective optimization algorithms were proposed, including the Pareto archive evolutionary strategy (PASE) [11,12], strength Pareto evolutionary algorithm (SPEA 2) [13], non-dominated sorting genetic algorithm II (NSGA-II) [14], non-dominated sorting particle swarm optimization (PSO) [15], and multi-objective particle swarm optimization (MOPSO) [16,17]. It has been shown that MOPSO achieved the highest optimization capacity and rate of convergence [18,19]. However, it is prone to the premature convergence of the MMIF process [20]. The authors in [20] proposed a new method, called the Fractional order Darwinian particle Swarm optimization (FODPSO), to escape from local optima, though its results could be improved.

To overcome the above-mentioned drawback, this paper proposes a variable-order fractional-order multi-objective Darwinian Particle Swarm Optimization combined with a gradient compass-based multimodal medical image fusion method (called VF-MODPSO-GC). The proposed approach fuses two types of multimodal medical images, that is, MRI and CT. This fusion ensures improved visual quality of the fused images and increases the speed of the fusion process. The proposed approach is carried out as follows: First, edge details are generated from the source medical images in eight varying directions. These edges provide relevant information for designing an edge map of the input medical images. The edge maps are then used to obtain two detailed medical images. The statistical features of the detailed medical images are then processed alongside the proposed multi-objective Darwinian Particle Swarm optimization to generate optimal weight matrices. Finally, pixel fusion is carried out between the two-source multimodal medical images. The proposed method is evaluated using a dataset generated from the Harvard Medical Image Database (https://dataverse.harvard.edu/). The principal contributions of this paper are as follows:

1. A novel optimization algorithm is introduced, combining the multi-objective Darwinian Particle Swarm Optimization with a variable-order fractional calculus operator (VF-MODPSO). The Variable-order Fractional-order (VF) contributes to enhancing the convergence rate of MODPSO.
2. The novel optimization algorithm is designed to address the issue of premature convergence commonly observed in multimodal MIF.
3. The proposed algorithm contributes to enhancing the feature extraction phase. This algorithm optimizes the weight matrices derived from the two detailed medical images produced during the gradient compass process of Multimodal MIF.
4. The proposed algorithm is applied using the gradient compass image generator. This composite model successfully achieves two primary objectives: maintaining high image quality during the fusion process and preserving intricate details from the source images in the final fused image.

The evaluation results show high performance in transferring sufficient details from source medical images to the fused medical images, while increasing the processing speed.

The rest of this paper is organized as follows: Section 2 presents the related works. Section 3 presents the background and the proposed technique. Section 4 provides the performance metrics. Section 5 presents the experiment results and the comparison study of VF-MODPSO with other optimization algorithms through a series of test instances and shows the application of the proposed algorithm to medical image fusion. Section 6 discusses the dynamics of the results. Finally, conclusions and future perspectives are drawn in Section 7.

## 2. Related works

This section reviews recent works in multimodal medical image fusion, particularly focusing on optimization algorithms, fractional calculus, and machine learning techniques. The goal is to identify the limitations in existing approaches and highlight the novelty of the proposed method.

Recently, the application of AI-based tools to improve diagnostic efficacy as well as healthcare assistance has become a very active area of research. Over the years, different medical image fusion methods have been proposed [21–23]. Moreover, the application of deep learning to image processing has gained considerable attention. Deep learning was introduced to medical image fusion while attempting to address the design challenge of activity level measurement and the fusion rule found in traditional methods [24].

In this section, we reviewed the recent state-of-the-art studies that applied AI algorithms to enhance the fusion process, along with existing works that utilized deep learning and neural network-based medical imaging techniques. In [23], authors developed a novel deep medical image fusion approach based on a deep convolutional neural network (DCNN) to directly learn image features from the original images. Specifically, they used a pre-trained CNN model to extract deep features from the principal components of the decomposed source images. The method was evaluated using Edge Strength Measure (QAB/F) and entropy on six pairs of CT and MRI images related to distinct brain diseases. The results showed a QAB/F of 0.704 with 6.23 entropy for normal brain images and 0.775 with 4.75 entropy for Alzheimer diseases. However, the deep learning-based components of the method may be prone to overfitting problems and have high training cost.

Medical image translation using a fully conditioned bounded deep network was conducted in [24]. Bayesian deep learning was applied [25,26] for the registration of noisy medical images affected by nonlinear geometric irregularities. Medical image denoising was conducted in [27] via a multilayer deep residue network combined with sectionalized dictionaries. In [28], deep cascade restructuring of low-grade reduced-resolution computed tomography images of the chest was performed to enhance and resolve CT chest images, making them more effective for monitoring lung health in COVID-19 patients. However, the speed of all these AI-based diagnostic processes was not taken into consideration or improved.

Various optimization algorithms have been applied in multimodal image fusion. [7] used the whale optimization algorithm to optimize PCNN. [8] utilized multi-source information encoding optimization. [9] applied wavelet transform and the XGboost optimization algorithm. [10] employed metaheuristic optimization. [11] used boosted grey wolf optimizer. [12] applied improved sand cat optimization. [13] applied multimetric routing protocol optimization.

Furthermore, optimization techniques have played an important role in improving the quality and speed of MMIF. Optimization algorithms have been utilized to enhance the fusion process by targeting specific aspects. Duan et al. [29] presented an MMIF fusion method that applies a genetic algorithm in the optimization of the extracted features from the log-Gabor filter and sum-modified Laplacian. The results of their work revealed good local and spatial information retention abilities that are not commonly found in pixel-based fusion methods. However, image brightness needs to be improved. Das et al. [30] presented an MMIF framework that applies gray wolf optimization in the optimization of decomposed cartoon and texture components from an optimized low-rank texture prior model. Their results showed good structural information preservation; however, image contrast still needs improvement.

Also, fractional calculus was incorporated into MMIF to improve convergence rates and feature extraction. Bhardwaj and Nayak [31] applied fractional bird swarm optimization for the optimization of the Bayesian approach used for the fusion of decomposed sub-bands. The results obtained showed fairly good visual quality of fused images; however, the edge-preservation ability suboptimal. Mergin and Premi [32] used neural networks with convolutional layers and the PSO algorithm with quantum behavior to develop a new approach for merging multimodal medical pictures. The results obtained showed excellent information retention ability; however, the speed of the fusion process was low. Kaur and Singh [33] applied multi-objective differential evolution to optimize the extracted features of decomposed sub-bands from source images for MMIF. The results obtained showed excellent edge preservation ability; however, the image brightness was not excellent. Prashantha and Prakash [34] presented a feature fusion method using a genetic algorithm and deep learning techniques. The results obtained showed good visual quality fusion image results; however, the edge-preservation ability was not good. Tang et al. [22] presented an MMIF method that applies a multi-swarm fruit fly optimization algorithm in the optimization of the hyperparameters of PCNN, resulting in fused images of good visual quality. However, the speed of the fusion process remained low. Das et al. [10] and [35] used differential evolution for the optimization of decomposed sub-bands obtained through deep neural networks and pulse-coupled neural networks, respectively, for MMIF. The results showed that the fused images preserved structural and textural details; however, image brightness needs improvement.

The reviewed studies indicate that several challenges still persist in MMIF, including slow fusion speeds, premature convergence, and difficulties in optimizing specific image features such as brightness and edge preservation. Our approach aims to address these issues by combining variable-order fractional calculus with multi-objective optimization.

## 3. Background and proposed method

In this section, we will start by presenting the different algorithms involved in our technique. Then, we will present our proposed method.

### 3.1. Background

First, we will present the gradient compass-based method. Then, we will give an overview about multi-objective optimization and Darwinian particle swarm optimization. Finally, we will discuss the variable-order fractional calculus operator.

#### The gradient compass-based method.

One approach to merging medical images from different modalities at the element level is the gradient compass-based method proposed in [36]. The gradient compass edge detection algorithm demonstrated good performance compared to other fusion techniques, such as wavelet transform, shearlet transform, and guided filtering as stated in [36]. The comparative analysis revealed the superior performance of the gradient-compass-based image fusion method across the diverse set of evaluation metrics. When compared to established techniques, including wavelet transform, shearlet transform, guided filtering, Laplacian Pyramid, Contourlet Transform, and a representative CNN-based fusion architecture, the gradient-compass method consistently achieved higher scores in metrics such as Peak Signal-to-Noise Ratio (PSNR), Structural Similarity Index (SSIM), and Mutual Information (MI) [36]. This indicates that the fused images generated by the gradient-compass approach exhibit improved visual quality, greater structural preservation, and a higher degree of information transfer from the source images. The gradient-compass method’s ability to effectively capture and integrate edge information from multiple input images appears to be a key factor in its improved performance, leading to more accurate and visually pleasing fusion results. The gradient compass-based method consists of four distinct steps: edge detection, detail image generation, weight generation, and pixel merging.

*Step 1: Edge detection*

**Fig 1:**
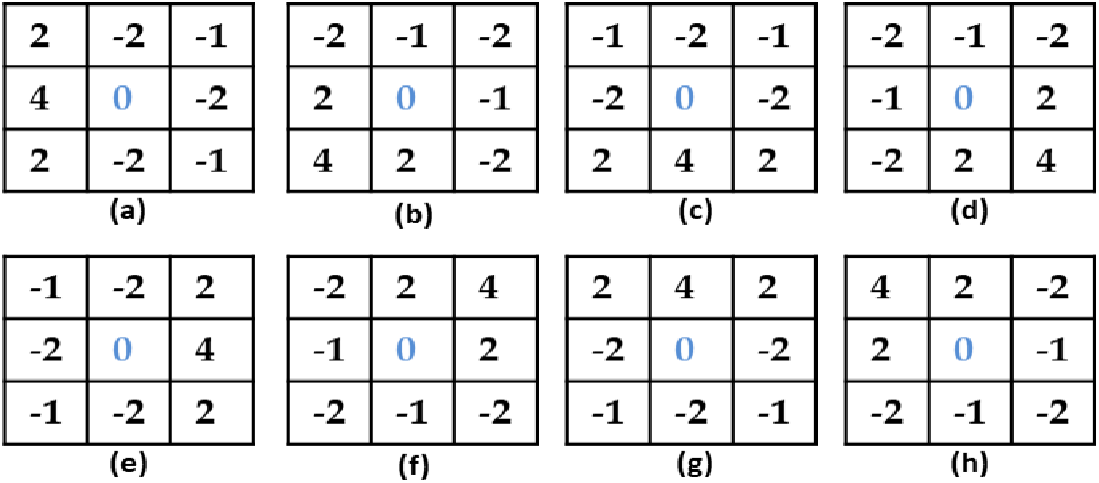
Compass gradients. **(a)**: GC 0˚, **(b)**: GC 45˚, **(c)**: GC 90˚, **(d)**: GC 135˚, **(e)**: GC 180˚, **(f)**: GC 225˚, **(g)**: GC 270˚, **(h)**: GC 315˚.

Medical images encompass several significant features. However, these features by themselves do not accurately outline the shape, structure, and boundaries of a specific organ. Also, they do not efficiently distinguish one organ from others, particularly when organs overlap, or edges are unclear [26]. Thus, the details of the medical image that indicate the edges are highly significant. Many techniques have been introduced to highlight boundaries in medical imaging, and every method consists of its own set of prerequisites [26]. One use of the gradient compass technique is the detection of edge information in input medical images using the Sobel gradient compass. It utilizes eight masks $\left(E_{0},E_{45},E_{90},E_{135},E_{180},E_{225},E_{270}andE_{315}\right)$, shown in Fig 1, each yielding boundary strength in any of the eight possible compass directions. Sobel gradient compass is renowned for preserving important information from the source images and preventing the addition of undesirable artifacts to the fused images [26]. The Sobel filter is among the most fundamental filters utilized for edge identification. It employs two 3x3 kernels, one for horizontal edges and another for vertical edges, which are convolved with the image. The kernels are designed to estimate the first-order derivatives of the image along the x and y axes, which quantify the gradient or the rate of change in pixel intensity. The Sobel filter subsequently integrates the horizontal and vertical gradients to derive the edge magnitude and direction. The Sobel filter offers the benefits of low complexity, straightforward implementation, rapid computation, and resilience to noise. It possesses drawbacks such as sensitivity to diagonal edges, the generation of thick edges, and an inability to consider edge continuity or smoothness; nonetheless, its advantages are very advantageous for multimodal medical picture fusion [36].

The computation of edge intensity at coordinates (x,y) is carried out using Eqn (1).


$$E_{\left(x,y\right)}=max\left(E_{(x,y)0},E_{(x,y)45},E_{(x,y)90},E_{(x,y)135},E_{(x,y)180},E_{(x,y)225},E_{(x,y)270},E_{(x,y)315}\right)$$

We select the output results of the gradient masks $E_{0},E_{45},E_{90},andE_{135}$ only because they are same as the reversals $E_{180},E_{225},E_{270},andE_{315}$. Then, we apply Eqn (1) on the source images $I_{1}andI_{2}$ as:


$${EI}_{1(x,y)}=max\left(\left|I_{1(x,y)0}\right|,\left|I_{1(x,y)45}\right|,\left|I_{1(x,y)90}\right|,\left|I_{1(x,y)135}\right|\right)$$ (2)

$${EI}_{2(x,y)}=max\left(\left|I_{2(x,y)0}\right|,\left|I_{2(x,y)45}\right|,\left|I_{2(x,y)90}\right|,\left|I_{2(x,y)135}\right|\right)$$ (3)

*Step 2: Detail image generation*

With the application of specific directional edge maps, two detailed images ${DI}_{1(x,y)}and{DI}_{2(x,y)}$ are obtained. The generated maximum edge strength maps, ${EI}_{1(x,y)}and{EI}_{2(x,y)}$ are deducted from the source medical scans $I_{2(x,y)}andI_{1(x,y)}$ to obtain detail images ${DI}_{1(x,y)}and{DI}_{2(x,y)}$.


$${DI}_{1(x,y)}=I_{2(x,y)}-{EI}_{1(x,y)}$$ (4)

$${DI}_{2(x,y)}=I_{1(x,y)}-{EI}_{2(x,y)}$$ (5)

Eqn (4) is applied to detect $I_{2(x,y)}$ peculiar details inherent in $I_{1(x,y)}$, and the peculiar details of image $I_{2(x,y)}$ are detected from image $I_{1(x,y)}$ through the detail image ${DI}_{2(x,y)}$ via Eqn (5).

*Step 3: Weight generation*

A statistical method is applied to obtain weights in the form of two weight matrices, ${wt}_{1}and{wt}_{2}$, using the detail images ${DI}_{1(x,y)}and{DI}_{2(x,y)}$. First, a window of size W×W is generated and passed through each element ${DI}_{1(x,y)}$. The region within it is specified as a matrix M, which is composed of W observations and variables, representing the rows and the columns. The matrix M is considered the vicinity of the corresponding component of *DI*_*1(x,y)*_. Next, the independent covariance matrix $C_{h}^{x,y}$ is computed from the matrix M using Eqn (6).


$$C_{h}^{x,y}=\frac{1}{W-1}\left[M^{T}M\right]$$ (6)

$$\begin{array}{c} {edgeStrength}_{h(x,y)}=\sum {\mathrm{\backslash nolimits}}_{i=1}^{W}eigenofC_{h}^{x,y}(i) \end{array}$$ (7)

$$\begin{array}{c} {edgeStrength}_{v(x,y)}=\sum {\mathrm{\backslash nolimits}}_{i=1}^{W}eigenofC_{v}^{x,y}(i) \end{array}$$ (8)

The sum of ${edgeStrength}_{h}$ and ${edgeStrength}_{v}$ is taken as the weight for one pixel $I_{1(x,y)}$ using Eq. (9).


$$weight(x,y)={edgeStrength}_{h(x,y)}+{edgeStrength}_{v(x,y)}$$ (9)

*Step 4: Pixel merging*

The merging of pixels marks the completion of the gradient compass method’s procedure. Adaptive image element fusion was applied in [26] during the pixel merging step.

#### Multi-objective optimization.

The subsequent definitions succinctly delineate the terms related to optimization of multiple objectives [36]:

**Multi-objective optimization:** A decision-making procedure that involves the consideration of multiple competing objectives. The aim is to identify a set of solutions that strike a balance between these objectives.


$$F(x)=\left(f_{1}(x),f_{2}(x),\ldots ,f_{m}(x)\right),$$

$$s.t.g_{i}(x)\leq 0,i=1,2,\ldots ,p,$$

$$h_{j}(x)=0,j=1,2,\ldots ,l.$$ (10)

Where $x=(x_{1},x_{2},\ldots ,x_{D})$ is a D-dimensional decision variable. *m* is the number of objective functions in the multi-objective optimization problem. $f_{m}(x$ represents the *m*^th^ objective optimization function. $g_{i}(xis$ the *i*^th^ inequality constraint, *p* the number of inequality constraints, $h_{j}(x$ represents the *j*^th^ equation constraint, and *l* is the number of equation constraints.

**Pareto dominance:** A solution A dominates a solution B if it is at least as excellent as B in all objectives and strictly better in one or more objectives. The two solutions in the feasible region $X_{\Omega }$ are represented as $x_{1},x_{2}$. $x_{1}$ can be seen as the solution that dominates $x_{2}$, and this relationship is mathematically denoted by $x_{1}>x_{2}$ if and only if


$$\forall i\in \left\{1,2,\ldots ,m\right\},f_{i}\left(x_{1}\right)\leq f_{i}\left(x_{2}\right),$$

$$\wedge \exists j\in \left\{1,2,\ldots ,m\right\},f_{j}\left(x_{1}\right)<f_{j}\left(x_{2}\right).$$ (11)

**Non-dominated solution**: A solution is non-dominated if there is no other solution in the population that dominates it.

**Pareto optimal solution:** A solution that is not controlled by an additional conceivable solution.

**Pareto Optimal Set** The collection of all Pareto optimal solutions. It is defined as:


$$P=\left\{x^{*}\in X_{\Omega }|\neg \exists x\in X_{\Omega }:x>x^{*}\right\}.$$ (12)

**Pareto Front:** The visual representation of the Pareto optimal set in the objective space. It is defined as:


PF={F(x)∣x∈P}. (13)

Typically, it is difficult to determine the exact shape of the Pareto front analytically. Instead, we aim to achieve three objectives: maximize the amount of non-dominated alternatives, guarantee a fair distribution of solutions throughout the Pareto front, and reduce the gap between the solutions produced by our method and the actual Pareto front, if one exists. Calculating the exact mathematical equation of the Pareto function is often impractical [36]. Therefore, we focus on three key goals: generating a significant quantity of non-inferior alternatives, minimizing the variation between our algorithm’s output and the ideal Pareto front (if its location is known), and ensuring that the generated solutions are evenly spread across the Pareto front.

#### Darwinian particle swarm optimization.

The Darwinian Particle Swarm Optimization (DPSO) algorithm is an enhanced version of the Particle Swarm Optimization (PSO) that includes elements of Darwinian principles, such as survival of the fittest, to improve the optimization process [37].

Darwinian Particle Swarm Optimization (DPSO) is highly effective for image fusion due to its ability to maintain diversity and avoid premature convergence through natural selection principles. By utilizing multiple swarms and retaining only the best-performing particles, DPSO ensures robust optimization and reliable convergence to high-quality solutions. This adaptability allows DPSO to handle the complex optimization landscapes of image fusion tasks, such as selecting the most relevant features or coefficients from source images. Additionally, its focus on survival-of-the-fittest mechanisms ensures that the fused image preserves maximum information, achieving superior fusion results compared to traditional methods [38].

The improvements to the particle swarm algorithm are generally given as follows: Eqn (14) and Eqn (15) below:


$$V_{t+1}^{n}=wV_{t}^{n}+\rho_{1}r_{1}\left({\bar{\text{g}}}_{t}^{n}-x_{t}^{n}\right)+\rho_{2}r_{2}\left({\dot{\text{x}}}_{t}^{n}-x_{t}^{n}\right)+\rho_{3}r_{3}\left({\tilde{\text{n}}}_{t}^{n}-x_{t}^{n}\right)$$ (14)

$$x_{t+1}^{n}=x_{t}^{n}+V_{t+1}^{n}$$ (15)

Where n is the moving particle and tis the time step. $x_{t}^{n}$ is the particle’s location. $V_{t}^{n}$ represents the particle’s velocity. ${\dot{\text{x}}}_{t}^{n}$ stands for each particle’s local best position. ${\tilde{\text{n}}}_{t}^{n}$ is the best position found within the neighborhood of the nth particle. ${\bar{\text{g}}}_{t}^{n}$ istheglobalbestposition. *ρ1, ρ2, and ρ3* prioritize the top performers on a global, regional, and neighborhood scale, respectively, when the new velocity is determined. $r_{1}$, $r_{2}$ and $r_{3}$ are uniformly distributed between 0 and 1. These random numbers introduce variability and randomness into the velocity update of each particle in the Darwinian Particle Swarm Optimization (DPSO) algorithm, allowing the particles to “explore” the search space in different ways.

The features that distinguish DPSO from PSO include the removal of outdated particles, the destruction of ineffective particles, the renewal of the swarm, and the creation of new particles. In DPSO, when a particle is removed, the number of removed particles, $N_{kill}$, increases gradually and approaches a threshold value. As particles are never removed in PSO, this number is always equal to zero. The probability of a particle being selected for reproduction or survival in DPSO is called the Selection Coefficient, ${SC}_{c}$, as described in Eq (16).


$${SC}_{c}\left(N_{kill}\right)={SC}_{c}^{\max}\left[1-\frac{1}{N_{kill}+1}\right]$$ (16)

Where ${SC}_{c}^{\max}$ is the maximum value that the selection coefficient can attain.

#### Variable-order Fractional calculus operator (VF).

Fractional calculus is a mathematical instrument that is indispensable for practical sciences is [39]. Fractional calculus has enhanced the performance of numerous procedures applied in simulation, curve estimation, sorting, pattern identification, boundary detection, authentication, equilibrium, autonomy, observability, and durability [40]. Some works, like [41] and [40], employed fractional calculus to boost the convergence rate of PSO. This was achieved by accelerating the convergence of both particle velocities and positions, both in standard PSO and DPSO. Fractional-order Darwinian particle swarm optimization (FODPSO) was also utilized by [20] for multilayer thresholding of medical pictures. These applications demonstrate that incorporating fractional calculus improves the exploration capabilities of the algorithm, which leads to better optimization performance. VFs were introduced to address the variable nature of most real-world systems [42]. The definition of VF in the Grünwald-Letnikov approach [43] is expressed as shown in Eq


$${\text{Ə}}_{f}^{\alpha (t)}f(t)={\underset{h\to 0^{+}}{\text{lim}}h}^{-\alpha (t)}\sum_{k=0}^{\infty }{\left(-1\right)}^{k}\frac{{\left(-1\right)}^{k}{\left(-\alpha (t)\right)}_{k}}{k!}f(t-kh)$$ (17)

Where (a)k=a(a+1)(a+2ldots(a+k−1) is called the Pochhammer symbol.

### 3.2. Materials and methods

**Fig 2:**
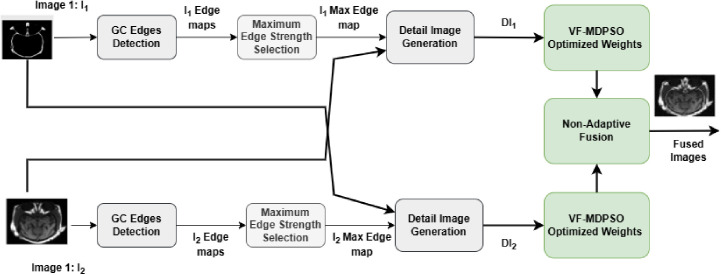
Schematic diagram of the proposed method. (GC: Gradient Compass).

The multimodal medical image fusion technique proposed in this study involves the merging of two input images of the same organ from various modalities. This is performed via a combination of three main techniques, including the proposed multi-objective optimization technique (MODPSO), the VF operator, and the gradient compass method in the spatial domain. In fact, VF is combined with MODPSO to enhance the premature convergence observed in MODPSO. In step 1, Gradient Compass Edge detection is applied to the two input images I_1_ and I_2_. In step2, specific chosen directional edge maps are applied to generate two detail images. ${DI}_{1(x,y)}and{DI}_{2(x,y)}$ Using Eqn. (1), boundary strength at location (x, y) is computed. The output results of the gradient compass mask $(E_{0},E_{45},E_{90},andE_{135}\backslash )$ are then chosen. The maximum edge strength maps and ${EI}_{1(x,y)}$${EI}_{2(x,y)}$ are then obtained via Eqn. (2) and Eqn. (3) and subtracted from the source images $I_{1(x,y)}$$I_{2(x,y)}$ to obtain detail images $I_{D1(x,y)}$$I_{D2(x,y)}$ according to Eqns. (4) and (5). In step3, the weight of a pixel $I_{1(x,y)}$$I_{2(x,y)}$ (or is obtained from Eqns. (6) – (9) with W=3,5,7,9; Therefore, we apply our VF-MODPSO algorithm to obtain the optimal weights $\left({weight}_{1(x,y)}and{weight}_{2(x,y)}\right)$ for the entire elements of the input images as well as $I_{1}$$I_{2}$, which are computed from their respective detailed scans according to Eqn. (9). Finally, in the fourth step, pixel fusion is carried out. Fig 2 represents a schematic diagram of the proposed method.

This section provides a comprehensive explanation of the proposed VF-MODPSO. The MODPSO algorithm is explained in Section 3.2.1. The VF approach is detailed in Section 3.2.2. Finally, the non-adaptive fusion technique is described in 3.2.3.

#### Multi-objective darwinian particle swarm optimization (MODPSO).

Drawing upon the principles of multi-objective optimization outlined in previous the section and the core concepts of the Darwinian Particle Swarm Optimization (DPSO) algorithm detailed also in the previous section, we suggest a new strategy for the MODPSO algorithm. The following are definitions of some of the algorithm’s fundamental terms:

- **Particle:** Within the framework of MODPSO, a particle serves as a potential solution to the multi-objective optimization challenge. Every particle possesses a coordinate vector that aligns with a particular set of decision variables, along with a vector of motion that defines both the trajectory and magnitude of the particle’s movement within the solution room.
- **Archive**: An archive is a repository of solutions that are not dominated and identified throughout the optimization procedure. It stores the best solutions discovered so far, helping to maintain diversity and direct the hunt towards the Pareto front.
- **Selection:** Selection in MODPSO typically relies on a combination of fitness and diversity. To this end, Pareto ranking is employed. This technique ranks solutions based on Pareto dominance, assigning higher ranks to non-dominated solutions. By applying Pareto ranking to the entire population, the algorithm prioritizes the selection of high-ranking solutions for reproduction. This strategy ensures that the search effort is directed towards the Pareto front, mitigating the risk of premature convergence to local optima. Pareto ranking offers several advantages: it preserves diversity within the population, facilitates efficient selection, and is well-suited for handling problems with multiple, conflicting objectives.
- **Objective Functions:** The target functions within a multiple objective optimization challenge represent the conflicting goals that need to be balanced. In our MODPSO algorithm, the objective functions are:
- **Minimize time:** This aims to reduce the running time of the convergence of particles.
- **Maximize fitness:** This aims to increase the fitness of the converging particles.
- Focusing on this research study, the objective is to enhance the quality of the merged image by optimizing factors including clarity, brightness, as well as information preservation. The goal is to generate a merged image that is both visually appealing and illuminating while minimizing artefacts and distortions. By carefully defining the objective functions and tuning the parameters of the MODPSO algorithm, we can effectively address multi-objective optimization problems. The fitness function is evaluated via Eqns. (18) and (19) below:

$${Fitness}_{1}=1-(ENT+FMI+Q_{abf})$$ (18)

$${Fitness}_{2}\propto 1/Time$$ (19)

Where ENT is entropy, which is applied to measure the extent of rich details in the fused image. FMI is a measure of the mutual dependence between the source image and the fused image. $Q_{abf}$ is the edge information similarity, which indicates the amount of edge information preserved from the source image that can be found in the fused image. Finally, Time is the running time of the fusion process. Eq (19) expresses an inverse relationship between fitness and time. As time decreases, fitness increases proportionally. This is a common scenario in optimization problems where the goal is to minimize time while maximizing performance or quality.

- **Termination criteria:** This is determined by the maximum number of iterations that will be implemented in the experiments.

**Algorithm 1:** MODPSO

**Step 1: Initialization** • Spontaneously set up an ensemble of particles, P. • Initialize the global best archive (gbest) and personal best archive (pbest) to empty sets. **Step 2: Selection** • Select the alternatives that are not dominated derived from the existing population, P, and revise the pbest repository. • Select the non-dominated solutions from the gbest archive and update the gbest archive. **Step 3: Update Velocities and Positions** • For every particle i in P: Revise the rate, *yi,* utilizing Eq (14) and Eq (16). Revise the position, *x*_*i*_, Eq (15) and Eq (16). **Step 4: Update Archives** • For each particle i in P: Add the particle’s’current position, *x*_*i*_, to the pbest archive. Remove dominated solutions from the pbest archive if necessary. • Add the alternatives that are not dominated from the current population to the gbest repository. • Remove dominated solutions from the gbest archive if necessary. **Step 5: Termination Check** • If the termination criteria are met, move on to Step 6. • Otherwise, return back to Step 2. **Step 6: Output** • Select the alternatives that are not dominated from the final gbest repository as the final set of Pareto optima					

#### Variable-order fractional-order based multi-objective darwinian particle swarm optimization (VF-MODPSO).

As discussed above, the goal of this combination is to enhance the convergence speed of MODPSO. For this, VF is incorporated in the Velocity equation. The fractional calculus derived from the Grünwald-Letnikof definition, which is derived from the principle of a fractional definition with a fractional coefficient ⍺ of a general signal *x(t),* is expressed by:


$$\begin{array}{c} D^{\propto }\left[x(t)\right]\left[\frac{1}{h^{\alpha }}\sum {\mathrm{\backslash nolimits}}_{k=0}^{+\infty }\frac{\left({-1)}^{k}\gamma (\alpha +1)x(t-kh\right)}{\gamma (k+1)\gamma (\alpha -k+1)}\right] \end{array}$$

where


γ (20)

is the gamma function

Equation (20) is for the time domain, but we will develop our novel method in the frequency domain. In the frequency domain, the fractional differential operator $S^{\alpha }$ may be estimated using the subsequent transfer function obtained from the Oustaloup method in the frequency domain (see Eq. (21)).


$$G_{\alpha_{i}}(S)=\frac{b_{\left[2N+1,\alpha_{i}\right]}S^{2N+1}+\ldots +b_{\left[1,\alpha_{i}\right]}S+b_{\left[0,\alpha_{i}\right]}}{a_{\left[2N+1,\alpha_{i}\right]}S^{2N+1}+\ldots +a_{\left[1,\alpha_{i}\right]}S+a_{\left[0,\alpha_{i}\right]}}$$ (21)

i=1,…,M−1

where N and M are integer constants, $a_{\left[2N+1,\alpha \right]}$,$a_{\left[2N,\alpha \right]},\ldots ,a_{\left[1,\alpha \right]}{,a}_{\left[0,\alpha \right]}$are related denominator coefficients, and $b_{\left[2N+1,\alpha \right]}$, $b_{\left[2N,\alpha \right]},\ldots ,b_{\left[1,\alpha \right]}{,b}_{\left[0,\alpha \right]}$are related numerator coefficients.

By applying the variable-order fractional calculus (VF) operator to the MODPSO algorithm, we obtain:


$$V_{t+1}^{n}=\frac{b_{\left[2N+1,\alpha_{i}\right]}{V_{t}^{n}}^{\left(2N+1\right)}+\ldots +b_{\left[1,\alpha_{i}\right]}V_{t}^{n}+b_{\left[0,\alpha_{i}\right]}}{a_{\left[2N+1,\alpha_{i}\right]}{V_{t}^{n}}^{\left(2N+1\right)}+\ldots +a_{\left[1,\alpha_{i}\right]}V_{t}^{n}+a_{\left[0,\alpha_{i}\right]}}+\rho_{1}r_{1}\left({\bar{\text{g}}}_{t}^{n}-x_{t}^{n}\right)+\rho_{2}r_{2}\left({\dot{\text{x}}}_{t}^{n}-x_{t}^{n}\right)+\rho_{3}r_{3}\left({\tilde{\text{n}}}_{t}^{n}-x_{t}^{n}\right)$$ (22)

i=1,…,M−1

$a_{\left[2N+1,\alpha \right]}$ and $a_{\left[2N,\alpha \right]},\ldots ,a_{\left[1,\alpha \right]}{,a}_{\left[0,\alpha \right]}$are ⍺ related denominator coefficients, which we denote as $A_{k}^{T}$. $b_{\left[2N+1,\alpha \right]}$ and $b_{\left[2N,\alpha \right]},\ldots ,b_{\left[1,\alpha \right]}{,b}_{\left[0,\alpha \right]}$ are ⍺ related numerator coefficients, which we denote as $B_{k}^{T}$. N and M are integer constants that can be taken as 2 and 20 [24], respectively.

$A_{k}^{T}$ is the transpose of the matrix $A=\left[A_{2n+1},\ldots ,A_{0}\right]$, and $B_{k}^{T}$ is the transpose of the matrix$B=\left[B_{2n+1},\ldots ,B_{0}\right]$ with k=0,1,…,2N+1.

For any given order α, its frequency domain transfer function can be gotten via the polynomial fitting technique via (${V_{t}^{n},A}_{k}^{T}$) and (${V_{t}^{n},B}_{k}^{T}$). Considering the five-order polynomial fitting, the polynomial fitting outcomes for the numerator coefficient and denominator coefficient are expressed in Eq 23 and Eq 24.


$${FB}_{k}(\alpha )=\varphi_{\left[5,k\right]}\alpha^{5}+\varphi_{\left[4,k\right]}\alpha^{4}+\ldots +\varphi_{\left[1,k\right]}\alpha +\varphi_{\left[0,k\right]}$$ (23)

k=2N+1,2N,…,2,1,0;

$${FA}_{k}(\alpha )=\underset{\left(denominator\right)}{\varphi_{\left[5,k\right]}\alpha^{5}+\varphi_{\left[4,k\right]}\alpha^{4}+\cdots +\varphi_{\left[1,k\right]}\alpha +\varphi_{\left[0,k\right]}}$$ (24)

k=2N+1,2N,…,2,1,0;

NB:Eqn. (22) is the main equation expressing the originality we added to MODPSO

We improved the MODPSO algorithm using VF to increase the convergence rate of MODPSO for image fusion processes. This is because we need an image fusion algorithm that produces high-quality fused images with complete information and negligible noise and will be useful to medical experts during real-time image-guided surgery. The overall structure of VF-MODPSO is outlined in Algorithm 2:

**Algorithm 2:** VF-MODPSO

1. **Step 1:Initialize** 2. Spontaneously create particles (*x*) in the possible region 3. gbest archive = *x* 4. pbest archive = *x*_*i*_, where *x*_*i*_ are particles in the *ith* generation 5. **Step 2:Choose gbest and pbest particles in the ***ith*** generation** 6. gbest = nondominated solutions selected from the gbest archive after extra solutions are removed 7. pbest_i_ = nondominated solutions selected from the pbest archive after extra solutions are removed 8. **Step 3:Modify ***x*****_***i***_*****(t)*** in accordance with** equations (14) and (22) 9. **Step 4:Update the gbest archive and pbest archive** 10. For i = 1: population size 11. pbestarchive(*t*) = nondominated selection (pbestarchive*(t-1), x*_*i*_*(t)*) 12. in the event that amount (pbestarchive(t)) exceeds the permitted capacity of the pbest archive 13. Eliminate the surplus particles within pbestarchive(*t*) 14. conclude 15. gbestarchive(*t*) = nondominated-sampling (gbestarchive(*t*)) 16. in the event that amount (gbestarchive(*t*))> population count 17. Eliminate the surplus particles within gbestarchive(*t*) 18. conclude 19. **Step 5:if the cessation criterion is met** 20. Termination 21. otherwise 22. Reiterate steps 2–5. 23. conclude 24. **Step 6:Choose the optimal alternative** 25. Retrieve the most suitable particle from the gbest archive by removing the extra solutions from the nondominated solutions if they are exceeding the specified quantity.					

#### Fusion step.

In this work, nonadaptive pixel fusion is introduced and applied. While adaptive pixel fusion applies new adaptive weights and noise-manipulating coefficients, nonadaptive pixel fusion applies optimized weight matrices and zero noise-manipulating coefficients. This makes our method less complex with no noise artefacts. Here, the merging procedure between the elements of input multimodal photos related to medicine, $I_{1}$ as well as $I_{2}$takes place via Eqn (25).


$$F_{\left(x,y\right)}=\frac{I_{1(x,y)}{weight}_{1(x,y)}+I_{2(x,y)}{weight}_{2(x,y)}}{{weight}_{1(x,y)}+{weight}_{2(x,y)}}$$ (25)

Image pixel of $I_{1(x,y)}$ and $I_{2(x,y)}$ possessing identical x and y vectors is merged sequentially to obtain the result of the fused medical image. In Eqn (14), $I_{1(x,y)}$and $I_{2(x,y)}$ represent actual pixels extracted from the input medical image, whereas ${weight}_{1(x,y)}and{weight}_{2(x,y)}$represent values of the weights derived from the corresponding weight arrays., ${wt}_{1}$ as well as the ${wt}_{2}$. $F_{\left(x,y\right)}$ stands for final merged element value, that consists of proportionate details from both multimodal source pixels.

## 4. Performance metrics

### 4.1. Quantitative metrics

In a multi-objective

optimization problem [36], the Pareto set (PS) comprises all Pareto optimum alternatives, while the Pareto front (PF) consists of the objective values associated with these Pareto optimal responses. The approximation set (AS) comprises nondominated alternatives derived from the search process, whereas its approximation front (AF) consists of the target variables corresponding to these nondominated solutions. When addressing multi-objective optimization problems, it is advantageous to possess two criteria. The AF approaches the PF as nearly as feasible to guarantee its precision. Secondly, AF disperses across the full PF with as much uniformity as feasible (variance) to produce a multitude of meaningful alternatives.

The solution of a multi-objective problem requires both convergence and diversity. In this work, we apply two metrics, Inverted Generational Distance (IGD) and Hyper-Volume (HV), as characteristic factors to evaluate the search performance of our VF-MODPSO algorithm. For more information about these metrics, the reader can refer to [44].

IGD measures how close is the solution to the PF. It can assess the convergence of multi-objective evolutionary algorithms during the resolution of multi-objective issue.

The HV can concurrently assess convergence and diversity by considering the coverage and distribution of solutions. It calculates the distance through reference locations and the participants in the AF.

This suggests that minimal IGD and elevated HV signify whether the AF has become proximate to the PF together with being dispersed as uniformly as feasible across the entire PF.

Therefore, IGD and HV are the two metrics used in this work to evaluate the effectiveness of VF-MODPSO-based GC and to compare it with other image fusion methods.

#### Mathematical expressions of the quantitative metrics.

1. **IGD**


$$IGD\left(A,P^{*}\right)=\frac{1}{\left|P^{*}\right|}\sum_{P\in P^{*}}{q\in A}^{d(p,q)}$$ (26)

Where:

- A: The approximation set, i.e., the set of solutions obtained by the algorithm.
- $P^{*}$: The true Pareto front, i.e., the reference set of optimal solutions.
- $\left|P^{*}\right|$: The number of points in the true pareto front.
- d(p,q): The distance between point p (from $P^{*}$) and q (from A), often computed using Euclidean distance:

$$d(p,q)=\sqrt{\sum_{i=1}^{m}{\left(p_{i}-q_{i}\right)}^{2}}$$ (27)

Where:

*m* is the number of objectives

2. **HV**

The hypervolume HV of set A is calculated as follows:


$$HV(A,r)=\int_{R(A,r)}dV$$ (28)

Where:

- A: The approximation set, i.e., the set of solutions obtained by the algorithm.
- r: A user-defined reference point.
- R(A,r): The region in the objective space dominated by A and bounded by r.
- dV: The infinitesimal volume element.

### 4.2. Qualitative metrics

The efficacy of the suggested strategy is assessed using many widely recognized evaluation indicators. The selected evaluation metrics are entropy (ENT), mutual information (MI), edge information similarity (Q*abf*), and the structural similarity index (SSIM) (for mathematical details, refer to [45–47]). Entropy (ENT) is utilized to quantify the degree of intricate details in the combined image. Mutual information (MI) quantifies the interdependence between the source image and the fused image. The edge detail similarity (Q*abf*) quantifies how much of the edge information retained from the original image is present in the combined image. The structurally equivalent index (SSIM) measures the fidelity of structural information from the input visuals retained in the combined picture. For all these indicators, elevated values signify superior performance. The duration of the fusion process for each comparable approach is also assessed. The shorter the runtime, the superior the method’s performance.

#### Mathematical expressions of qualitative metrics.

1. **ENT**


$$ENT=-\sum \left(P(x)*\log_{2}^{\left(P(x)\right)}\right)$$ (29)

Where:

- P(x) is the probability of occurrence of the event x.
- Σ represents the summation over all possible events.
- log2 is the base-2 logarithm.

2. **MI**

The formula for Mutual Information (MI) between two random variables *x and y* is:


MI(x;y)=ENT(x)−ENT(y) (30)

Where:

- MI(x;y) represents the Mutual Information between *x and y*.
- ENT(x) is the entropy of *x*.
- ENT(y) is the conditional entropy of *x* given *y.*

3. **SSIM**


$$SSIM(F,I)=\frac{\left(\left(2\mu_{F}\mu_{I}\times C_{1}\right)\times \left(2\sigma_{FI}+C_{2}\right)\right)}{\left(\left(\mu_{F}^{2}+\mu_{I}^{2}+C_{1}\right)\left(\sigma_{F}^{2}+\sigma_{I}^{2}+C_{2}\right)\right)}$$ (31)

Where:

- F is the fused image
- I is the input image
- $\mu_{F}and\mu_{I}$ are the mean intensity of image F and I respectively
- $\sigma_{F}$ and $\sigma_{I}$ stand for the variance of images F and I respectively
- $\sigma_{FI}$ computes the covariance if images F and I
- $C_{1}$and $C_{2}$ are constants

## 5. Results and discussion

### 5.1. Experimental configuration

**Fig 3:**
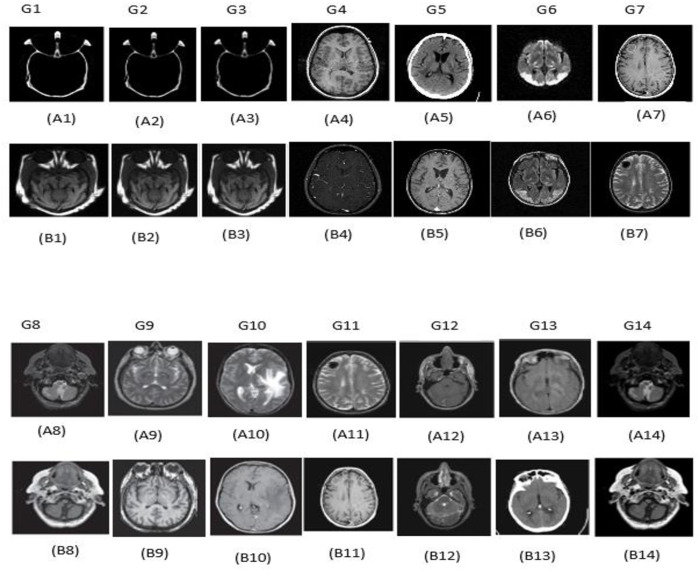
Source images from Group 1 (G1) to Group 14 (G14).

To conduct our experiments, fourteen groups of medical images (CT and MR scans) are utilized to assess the precision of the proposed approach. Each medical image group consists of two images. In total, twenty-eight images were deployed as a dataset for our experiments. These can be seen in Fig 3. Images A represent the input CT images, and images B represent the input MR images, which will be fused.

The images are 256×256 pixel resolutions. All the images in the dataset are correctly pre-registered. The source images can be found in http://www.med.harvard.edu/aanlib/. The MATLAB software is used in this simulation with the following specifications, R2018a version 9.4.0.813654, 64 bits, and the hardware 3.20 GHz, 4 GB of RAM, and Windows 7.

To assess the efficacy of the suggested strategy (VF-MODPSO-based MMIF), the parameters are set according to the recommendations mentioned in the related works [8–10]. Some comparative experiments were conducted with eight other image fusion algorithms on five test instances $f_{1}-f_{5}$ [40,41], using some performance metrics. The test functions are the optimization functions usually deployed during the tests of multi-objective optimization algorithms. These optimization functions consist of minimizing several well-known functions namely: Bohachevsky 1, Colville, Drop wave, Easom, and Rastrigin. More details can be found in [40,41]. The eight image fusion algorithms include GA + LGF-SML [7], GWO+OLTP [8], FBSO+HWD [9], QPSO+CNN [10], DE + DNN [11], GA + DL-CNN [12], MFOA+PCNN [13], and DE+PCNN [35]. All the compared algorithms were implemented. Termination criteria of the proposed optimization algorithm are similar to those used in [36]: Maximum Iterations: 1000; Hypervolume Convergence Threshold: 0.01; Population Diversity Threshold: 0.2; Time Limit: 3600 seconds (1 hour). The fractional-order multi-objective Darwinian particle swarm optimization (FOMODPSO) is an advancement of the fractional-order Darwinian particle swarm optimization (FODPSO), which itself is an extension of the standard Darwinian particle swarm optimization (DPSO) [40]. The most efficient results of VF-based DPSO are obtained by varying the values of the fractional coefficient (⍺) [24]. Tuning the parameter is essential in obtaining robust results. The greater the order of the fractional coefficients, the faster the convergence rate is. In this work, the order is varied within the range [0.6–10] [43].

### 5.2. Experimental results

#### Quantitative evaluation results and analysis.

The basis points for computing the HV in the subsequent experiments are established as follows:


$$z={\left(2,2\right)}^{T},z={\left(2,2,2\right)}^{T},z={\left(2,2,2\right)}^{T},z={\left(2,2\right)}^{T},z={\left(2,3\right)}^{T}forf_{1}-f_{5}$$ (32)

The proposed method is tested many times. The experiments yielded stable (unchanged) results when the number of runs reached 31. So, this number was set to 31. The IGD and HV (mean and standard deviation) measurements of the merged images for each of the test instances $f_{1}-f_{5}$ have been calculated and are presented in Table 1. Table 1 displays the results of VF-MODPSO + GC and other image fusion methods. The initial quality indicators presented in Table 1 represent the mean, with the standard deviation for every one of the IGD and HV indicated at the right side of the mean. Rankings are displayed in brackets in square format adjacent to the quality measurements on the right side. To indicate the outcome of the comparison of VF-MODPSO + GC with the referenced method, we utilize the symbols “˫” for better performance, “≬” for worse performance, and “∾” for similar performance. The mean ranking (MR) is derived from each method across all test instances. The counts of “˫”, “≬”, and “∾” for every contrasted method are calculated to assess the typical efficiency of the image fusion methods.

**Table 1:** Obtained results (mean (std. dev.)[rank]) of the IGD and HV metric values of the proposed VF-MODPSO + GC and eight state-of-the-art image fusion algorithms.

Instance	GA + LGF-SML	GWO+OLTP	FBSO+HWD	QPSO+CNN	DE + DNN
IGD					
f1	6.246e-02^˫^(5.79e-04) [8]	6.550e-02^˫^(4.89e-04) [9]	5.931e-02^˫^(6.55e-03) [7]	7.786e-03^˫^(5.09e-04) [6]	6.974e-03^˫^(4.89e-03) [3]
f2	6.834e-02^˫^(6.72e-02) [9]	5.294e-02^˫^(6.90e-04) [7]	4.436e-02^˫^(6.78e-03) [6]	5.634e-02^˫^(7.43e-03) [8]	9.634e-03^˫^(5.44e-03) [5]
f3	7.224e-02^˫^(3.86e-03) [8]	5.626e-02^˫^(7.74e-03) [6]	8.278e-03^˫^(4.22e-03) [9]	6.913e-02^˫^(2.17e-05) [7]	9.547e-03^˫^(1.43e-04) [5]
f4	4.653e-02^˫^(1.42e-04) [9]	8.941e-03^˫^(3. [49]e-03) [7]	7.258e-03^˫^(9.06e-05) [5]	4.527e-02^˫^(3.34e-03) [8]	5.430e-04^∾^(5.84e-05) [2]
f5	5.662e-02^˫^(5.89e-03) [7]	6.458e-02^˫^(2.75e-03) [8]	6.953e-02^˫^(6.73e-04) [9]	5.198e-02^˫^(5.57e-03) [6]	9.267e-03^˫^(4.78e-03) [4]
HV					
f1	1.639e + 01^˫^(3.78e-03) [7]	1.610e + 01^˫^(5.45e-03) [8]	1.543e + 01^˫^(3.54e-03) [9]	1.697e + 01^˫^(1.88e-03) [5]	1.655e + 01^˫^(5.89e-03) [6]
f2	2.147e + 00^˫^(3.32e-04) [9]	2.551e + 00^˫^(5.67e-03) [6]	2.382e + 00^˫^(5.96e-03) [7]	2.749e + 00^˫^(2.70e-03) [4]	2.335e + 00^˫^(4.85e-03) [8]
f3	2.457e + 00^˫^(7.37e-04) [8]	2.223e + 00^˫^(6.47e-04) [9]	2.752e + 00^˫^(5.34e-04) [7]	2.892e + 00^˫^(6.86e-03) [6]	3.151e + 00^˫^(1.62e-03) [4]
f4	3.171e + 00^˫^(5.23e-03) [9]	3.876e + 00^˫^(2.35e-03) [7]	4.014e + 00^˫^(7.64e-03) [6]	3.537e + 00^˫^(5.09e-03) [8]	4.757e + 00^∾^(7.04e-04) [2]
f5	4.178e + 00^˫^(2.07e-03) [8]	4.357e + 00^˫^(3.97e-03) [6]	4.113e + 00^˫^(3.54e-03) [9]	4.323e + 00^˫^(7.54e-03) [7]	4.381e + 00^˫^(6.75e-04) [5]
Mean rank	8.200	7.300	7.400	6.500	4.400
˫/≬/∾	10/0/0	10/0/0	10/0/0	10/0/0	8/0/2
Instance	GA + DL-CNN	MFOA+PCNN	DE+PCNN	VF-MODPSO + GC	
IGD					
f1	7.532e-03^˫^(8.32e-03) [5]	7.368e-03^˫^(7.55e-03) [4]	6.657e-03^˫^(8.56e-05) [2]	6.484e-03(2.36e-04) [1]	
f2	8.984e-03^˫^(4.67e-03) [3]	9.242e-03^˫^(7.54e-04) [4]	8.852e-03^≬^(3.68e-03) [1]	8.897e-03(5.59e-04) [2]	
f3	8.853e-03^˫^(3.24e-04) [3]	8.652e-03^˫^(6.26e-03) [2]	9.140e-03^˫^(8.25e-04) [4]	8.651e-03(6.56e-04) [1]	
f4	8.703e-03^˫^(6.30e-04) [6]	6.902e-03^˫^(3.41e-04) [4]	6.453e-03^˫^(6.25e-03) [3]	5.429e-04(2.57e-04) [1]	
f5	9.838e-03^˫^(7.16e-03) [5]	8.936e-03^˫^(5.63e-03) [3]	8.748e-03^˫^(5.85e-04) [2]	8.648e-03(5.47e-05) [1]	
HV					
f1	1.764e + 01^˫^(2.38e-03) [3]	1.715e + 01^˫^(2.89e-03) [4]	1.748e + 01^˫^(1.59e-04) [2]	1.808e + 01(7.78e-04) [1]	
f2	2.662e + 00^˫^(3.48e-03) [5]	2.873e + 00^˫^(3.05e-03) [2]	2.864e + 00^˫^(5.38e-04) [3]	2.9 [49]e + 00(8.69e-03) [1]	
f3	3.020e + 00^˫^(1.41e-03) [5]	3.667e + 00^˫^(7.31e-04) [2]	3.324e + 00^˫^(6.99e-03) [3]	3.668e + 00(4.75e-04) [1]	
f4	4.581e + 00^˫^(6.65e-04) [3]	4.258e + 00^˫^(4. [49]e-04) [5]	4.282e + 00^˫^(4.87e-03) [4]	4.757e + 00(6.32e-05) [1]	
f5	4.399e + 00^˫^(9.42e-04) [4]	4.615e + 00^˫^(6.68e-04) [3]	4.716e + 00^∾^(3.90e-04) [2]	4.874e + 00(7.98e-05) [1]	
Mean Rank	4.200	3.300	2.600	1.100	
˫/≬/∾	10/0/0	10/0/0	9/1/0		

**Table 3:** Various approaches and their fusion processing times (in seconds).

	Method								
Metric	GA + LGF-SML	GWO+OLTP	FBSO+ HWD	QPSO+ CNN	DE + DNN	GA + DL-CNN	MFOA+ PCNN	DE+ PCNN	VF-MODPSO+GC
TIME	5.530	5.150	3.430	2.590	1.990	0.750	1.260	0.390	**0.085**

#### Qualitative evaluation results and analysis.

To perform the qualitative evaluation, a Support Vector Machine (SVM) algorithm was employed. It is chosen for its effectiveness in handling high-dimensional data and achieving robust performance in binary classification tasks. The classification is performed to show whether the fused image exhibits high quality (Class 1) or low quality (Class 0). To create the labeled dataset, fused images were assigned to Class 1 if they achieved a $F_{1}$ score above 0.5 and exhibited no visible artifacts as determined by visual inspection by two independent reviewers. Fused images were assigned to Class 0 if they had $F_{1}$score below 0.5 or showed significant blurring or misalignment. Disagreements between reviewers were resolved through discussion and consensus. About 66.67% of the extracted dataset was used for training and 33.33% for validation. The image features that are used as input for the SVM include edge orientation, edge magnitude, edge density, and texture features. The SVM classifier employed an RBF kernel with hyperparameters (C and gamma) optimized using grid search with 3-fold cross-validation. The optimal hyperparameter values were found to be C = 1.0 and gamma = 0.01.

The accuracy, sensitivity, specificity, and F1 score are chosen as the assessment metrics to evaluate the efficiency of the suggested approach against the state-of-the-art methods used in the previous comparative study.

The experimental results for each of the cross-validation stages are shown in Table 2, while the detailed experimental outcomes of all the other stages are made available at:

**Table 2:** Performance evaluation via 3-fold cross-validation.

Method	Evaluation Parameter	K = 1	K = 2	K = 3	Average
	Accuracy	0.525	0.449	0.455	0.475
	Sensitivity	0.510	0.443	0.447	0.497
GA + LGF-SML	Specificity	0.733	0.652	0.657	0.681
	$F_{1}$ Score	0.518	0.440	0.443	0.494
	Accuracy	0.668	0.545	0.526	0.576
	Sensitivity	0.625	0.524	0.524	0.568
GWO+OLTP	Specificity	0.734	0.641	0.643	0.673
	$F_{1}$ Score	0.618	0.531	0.531	0.557
	Accuracy	0.662	0.559	0.539	0.580
	Sensitivity	0.638	0.530	0.538	0.562
FBSO+HWD	Specificity	0.735	0.731	0.721	0.739
	$F_{1}$ Score	0.613	0.535	0.534	0.561
	Accuracy	0.662	0.553	0.533	0.574
	Sensitivity	0.632	0.534	0.532	0.566
QPSO+CNN	Specificity	0.735	0.730	0.723	0.739
	$F_{1}$ Score	0.615	0.535	0.535	0.535
	Accuracy	0.679	0.557	0.548	0.698
	Sensitivity	0.635	0.548	0.537	0.570
DE + DNN	Specificity	0.737	0.732	0.726	0.732
	$F_{1}$ Score	0.628	0.549	0.548	0.565
	Accuracy	0.671	0.560	0.540	0.680
	Sensitivity	0.648	0.543	0.543	0.575
GA + DL-CNN	Specificity	0.634	0.748	0.730	0.748
	$F_{1}$ Score	0.621	0.543	0.543	0.579
	Accuracy	0.672	0.560	0.541	0.681
	Sensitivity	0.641	0.545	0.545	0.577
MFOA+PCNN	Specificity	0.755	0.749	0.736	0.743
	$F_{1}$ Score	0.624	0.546	0.545	0.572
DE + PCNN	Accuracy	0.674	0.562	0.541	0.682
	Sensitivity	0.644	0.558	0.559	0.580
	Specificity	0.777	0.762	0.740	0.760
	$F_{1}$ Score	0.638	0.558	0.558	0.575
Proposed	Accuracy	0.876	0.765	0.745	0.895
	Sensitivity	0.746	0.652	0.655	0.684
	Specificity	0.786	0.763	0.742	0.764
	$F_{1}$ Score	0.738	0.650	0.653	0.677

https://github.com/GoddessChysomme/FOMODPSO/issues/1

https://github.com/GoddessChysomme/FOMODPSO/issues/2

Moreover, the codes used in this work are open-sourced and can be found at:

https://github.com/GoddessChysomme/FOMODPSO/blob/main/fusion

https://github.com/GoddessChysomme/FOMODPSO/blob/main/xfusmaxmin

https://github.com/GoddessChysomme/FOMODPSO/blob/main/xfusmean

The in-depth analysis reveals that our proposed method yields the best fusion results with an average accuracy of 0.895, a sensitivity of 0.684, a specificity of 0.764, and an F_1_ score of 0.677.

## 6. Discussion

Table 1 proves that the suggested VF-MODPSO + GC method performs better than the other methods using the suggested metrics. Specifically, VF-MODPSO + GC has **79 instances where its mean metric values are better than the other methods** and **9 instances where it has the best (top) mean metric values among all methods**. This indicates a high-quality performance in achieving superior fused images. Additionally, VF-MODPSO + GC consistently achieved the best mean rank (MR), except on $f_{2}$ using IGD values, where DE+PCNN performed better. Based on the MR, the image fusion methods are ranked as follows: VF-MODPSO + GC, DE+PCNN, MFOA+PCNN, GA + DL-CNN, DE + DNN, QPSO+CNN, FBSO+HWD, GWO+OLTP, and GA + LGF-SML. Among all the image fusion methods, VF-MODPSO + GC exhibits outstanding efficiency on the test instances based on the measures of variety (search quality) and convergence.

**Fig 4:**
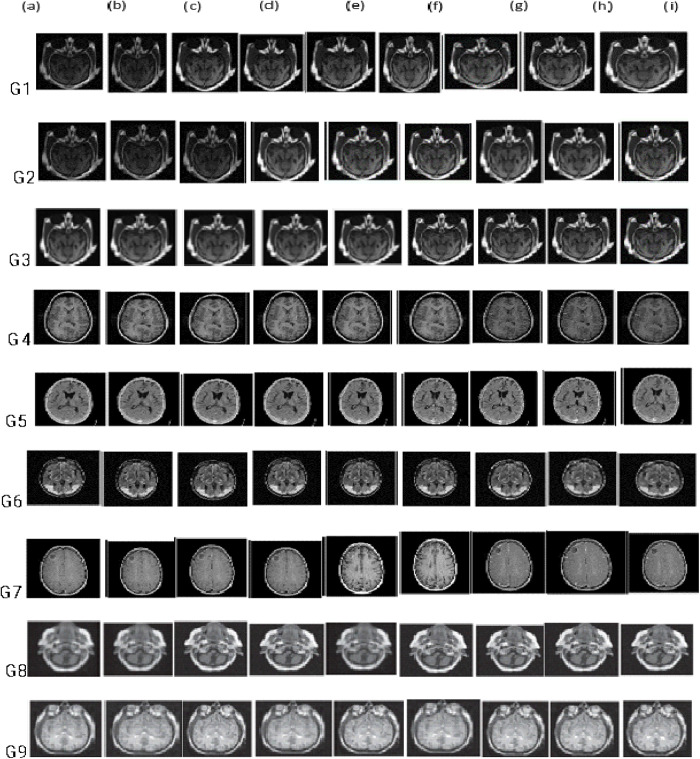
Medical image fusion results for (a) GA + LGF-SML (b) GWO+OLTP (c) FBSO+HWD (d) QPSO+CNN (e) DE + DNN (f) GA + DL-CNN (g) MFOA+PCNN (h) DE+PCNN (i) VF-MODPSO+GC.

The image fusion results of various multi-objective evolutionary approaches, including the suggested strategy, on fourteen groups of multimodal medical images are represented in Fig 4. The GA + LGF-SML approach has good image detail preservation ability; however, the brightness of the fused images needs to be determined. The GWO+OLTP multimodal image fusion method has excellent image structural detail preservation ability; however, there is minimal contrast in the fusion outcomes. The FBSO+HWD method has good visual quality fused image results; however, edge preservation is not good. The QPSO+CNN multimodal image fusion method has good information retention ability; however, the image brightness can be improved. The DE + DNN method shows excellent edge preservation; however, the fused image results are not bright enough.

The GA + DL-CNN method yields good visual quality fused images, but edge preservation is not as good. The MFOA+PCNN multimodal image fusion method yields good visual quality fused image results; however, the edge preservation ability needs to be improved.

The DE+PCNN yields fused image results with well-preserved structural and textural details; however, the image brightness needs improvement. **Fig 4** shows that the proposed fusion method gives fused image results of very good visual quality, efficient edge preservation ability, and no trace of noisy artefacts.

Furthermore, the speed of the fusion process with each comparative method was also evaluated and contrasted with the speed of the fusion process of the proposed VF-MODPSO + GC method, as shown in Table 3. Our method has a shorter run time than the other comparative approaches, namely, the DE+PCNN and GA + DL-CNN methods. The reason is that our method has low time consumption due to the increased optimization rate of the weights of the fusion rules. In fact, increasing the convergence rate of multi-objective Darwinian particle swarm optimization via a variable-order fractional calculus operator and applying the improved optimization algorithm to the weights of the fusion rules of the gradient compass in the spatial domain reduces the average time consumption of our method compared with those of other comparative methods.

Although its high performance, we recommend analyzing the scalability of the proposed method in terms of image modalities that are fused, as well as the size and depth of the input images. The current experiments involve a very small dataset of 14 2D images with only two modalities—CT and MRI. We recommend extending the numerical experiments to new datasets with more image modalities, larger images, and both 2D and 3D images (such as PET, SPECT, and ultrasound) to assess the robustness of the approach. One potential limitation of the proposed VF-MODPSO method is its sensitivity to high-dimensional data, which may lead to increased computational complexity and processing time, potentially affecting real-time applications. Additionally, the performance of the method may degrade when dealing with highly heterogeneous image modalities that exhibit significant differences in contrast, resolution, or noise levels. The fusion process could also be influenced by parameter tuning, where suboptimal choices may result in poor convergence or loss of critical image details. Further investigations on adaptive parameter selection and computational efficiency improvements, such as parallel processing or hardware acceleration, could help mitigate these challenges and ensure broader applicability of the method.

## 7. Conclusions

The field of medical imaging is considered incomplete without MMIF, as medical experts and researchers usually need multimodal fusion results for clinical diagnoses, treatment planning, and various medical studies. This study proposed a new method for MMIF that relies on the VF-MODPSO-based GC in the spatial domain. We first extracted edge details from source images in eight different directions in conjunction with a Sobel gradient compass. Then, the constructed edge maps are used to obtain two high-resolution medical pictures. We applied the comprehensive medical data’s statistical characteristics to build weight matrix systems, and then we used them to perform the merging. For the optimization of weight matrices, we proposed variable-order fractional-order multi-objective Darwinian particle swarm optimization. The improved convergence rate of VF-MODPSO contributed to enhancing the suggested method’s search efficiency. To evaluate the proposed approach, fourteen sets of CT/MR scans were used. In addition, the qualitative, visual, and quantitative evaluation metrics were employed to show how well the suggested method performs. Furthermore, the comparison study is achieved using eight multi-objective evolution-based MMIF methods.

The results showed the superiority of our method over the other methods in terms of search performance, visual quality, and fusion performance parameters. The proposed VF-MODPSO method could be adapted for video fusion.

## References

[1] Basu S, Singhal S, Singh D. A Systematic Literature Review on Multimodal Medical Image Fusion. Multimed Tools Appl. 2023;83(6):15845–913. doi: 10.1007/s11042-023-15913-w

[2] A. S, E. F. A survey on deep learning techniques for medical image fusion. IJEIT. 2024;12(1):7–16. doi: 10.36602/ijeit.v12i1.471

[3] Khan SU, Ullah I, Ullah N, Shah S, Affendi ME, Lee B. A novel CT image de-noising and fusion based deep learning network to screen for disease (COVID-19). Sci Rep. 2023;13(1):6601. doi: 10.1038/s41598-023-33614-0 37088788 PMC10122759

[4] Ghandour C, El-Shafai W, El-Rabaie E-SM, Elshazly EA. Applying medical image fusion based on a simple deep learning principal component analysis network. Multimed Tools Appl. 2023;83(2):5971–6003. doi: 10.1007/s11042-023-15856-2

[5] Behrouzi Y, Basiri A, Pourgholi R, Kiaei AA. Fusion of medical images using Nabla operator; Objective evaluations and step-by-step statistical comparisons. PLoS One. 2023;18(8):e0284873. doi: 10.1371/journal.pone.0284873 37585476 PMC10431637

[6] Dong L, Wang J, Zhao L, Zhang Y, Yang J. ICIF: Image fusion via information clustering and image features. PLoS One. 2023;18(8):e0286024. doi: 10.1371/journal.pone.0286024 37531364 PMC10396002

[7] Raha R, Sengupta A, Dhabal S. Medical Image Fusion using PCNN Optimized by Whale Optimization Algorithm. In: 2020 IEEE 1st International Conference for Convergence in Engineering (ICCE). IEEE; 2020. 374–8. doi: 10.1109/icce50343.2020.9290504

[8] Nie R, Cao J, Zhou D, Qian W. Multi-source information exchange encoding with PCNN for medical image fusion. IEEE Trans Circuits Syst Video Technol. 2021;31(3):986–1000. doi: 10.1109/tcsvt.2020.2998696

[9] Naseem S, Mahmood T, Khan AR, Farooq U, Nawazish S, Alamri FS, et al. Image Fusion Using Wavelet Transformation and XGboost Algorithm. CMC. 2024;79(1):801–17. doi: 10.32604/cmc.2024.047623

[10] Das M, Gupta D, Radeva P, Bakde AM. Multi‐scale decomposition‐based CT‐MR neurological image fusion using optimized bio‐inspired spiking neural model with meta‐heuristic optimization. Int J Imaging Syst Tech. 2021;31(4):2170–88. doi: 10.1002/ima.22575

[11] Zhang H, Cai Z, Xiao L, Heidari AA, Chen H, Zhao D, et al. Face image segmentation using boosted grey wolf optimizer. Biomimetics (Basel). 2023;8(6):484. doi: 10.3390/biomimetics8060484 37887615 PMC10604473

[12] Yao L, Yang J, Yuan P, Li G, Lu Y, Zhang T. Multi-strategy improved sand cat swarm optimization: global optimization and feature selection. Biomimetics (Basel). 2023;8(6):492. doi: 10.3390/biomimetics8060492 37887623 PMC10604673

[13] Ghorai C, Shakhari S, Banerjee I. A SPEA-Based Multimetric Routing Protocol for Intelligent Transportation Systems. IEEE Trans Intell Transport Syst. 2021;22(11):6737–47. doi: 10.1109/tits.2020.2994362

[14] Zhu J, Wang X, Huang H, Cheng S, Wu M. A NSGA-II Algorithm for Task Scheduling in UAV-Enabled MEC System. IEEE Trans Intell Transport Syst. 2022;23(7):9414–29. doi: 10.1109/tits.2021.3120019

[15] Li L, Chang L, Gu T, Sheng W, Wang W. On the Norm of Dominant Difference for Many-Objective Particle Swarm Optimization. IEEE Trans Cybern. 2021;51(4):2055–67. doi: 10.1109/TCYB.2019.2922287 31380777

[16] Xu L, Muhammad A, Pu Y, Zhou J, Zhang Y. Fractional-order quantum particle swarm optimization. PLoS One. 2019;14(6):e0218285. doi: 10.1371/journal.pone.0218285 31220152 PMC6586292

[17] Amir F, Farajzadeh A, Alzabut J. An improved proximal method with quasi-distance for nonconvex multiobjective optimization problem. J Appl Anal. 2022;28(2):333–40. doi: 10.1515/jaa-2021-2074

[18] Du S, Fan W, Liu Y. A novel multi-agent simulation based particle swarm optimization algorithm. PLoS One. 2022;17(10):e0275849. doi: 10.1371/journal.pone.0275849 36227927 PMC9560124

[19] Ghafour K. Multi-objective continuous review inventory policy using MOPSO and TOPSIS methods. Computers & Operations Research. 2024;163:106512. doi: 10.1016/j.cor.2023.106512

[20] Ahilan A, Chandra Babu G, Senthil Murugan N, Parthasarathy, Manogaran G, Raja C, et al. Segmentation by fractional order darwinian particle swarm optimization based multilevel thresholding and improved lossless prediction based compression algorithm for medical images. IEEE Access. 2019;7:89570–80. doi: 10.1109/access.2019.2891632

[21] Yang Y, Cao S, Huang S, Wan W. Multimodal medical image fusion based on weighted local energy matching measurement and improved spatial frequency. IEEE Trans Instrum Meas. 2021;70:1–16. doi: 10.1109/tim.2020.304691133776080

[22] Tang L, Tian C, Xu K. Exploiting quality-guided adaptive optimization for fusing multimodal medical images. IEEE Access. 2019;7:96048–59. doi: 10.1109/access.2019.2926833

[23] Kumar M, Ranjan N, Chourasia B. Hybrid Methods of Contourlet Transform and Particle Swarm Optimization for Multimodal Medical Image Fusion. In: 2021 International Conference on Artificial Intelligence and Smart Systems (ICAIS). 2021. 945–51. doi: 10.1109/icais50930.2021.9396021

[24] Zhang B, Jiang C, Hu Y, Chen Z. Medical Image Fusion Based a Densely Connected Convolutional Networks. In: 2021 IEEE 5th Advanced Information Technology, Electronic and Automation Control Conference (IAEAC), 2021. 2164–70. doi: 10.1109/iaeac50856.2021.9390712

[25] Challa UK, Yellamraju P, Bhatt JS. “A Multi-class Deep All-CNN for Detection of Diabetic Retinopathy Using Retinal Fundus Images,” 2019, pp. 191–9. doi: 10.1007/978-3-030-34869-4_21

[26] Irshad MT, Rehman HU. Gradient compass-based adaptive multimodal medical image fusion. IEEE Access. 2021;9:22662–70. doi: 10.1109/access.2021.3054843

[27] Rai S, Bhatt JS, Kumar Patra S. A Strictly Bounded Deep Network for Unpaired Cyclic Translation of Medical Images. In: 2023 IEEE Statistical Signal Processing Workshop (SSP). 2023;61–5. doi: 10.1109/ssp53291.2023.10207960

[28] Deshpande VS, Bhatt JS. Bayesian Deep Learning for Deformable Medical Image Registration. Lecture Notes in Computer Science. Springer International Publishing. 2019. p. 41–9. doi: 10.1007/978-3-030-34872-4_5

[29] Duan J, Mao S, Jin J, Zhou Z, Chen L, Chen CLP. A novel GA-based optimized approach for regional multimodal medical image fusion with superpixel segmentation. IEEE Access. 2021;9:96353–66. doi: 10.1109/access.2021.3094972

[30] Das M, Gupta D, Radeva P, Bakde AM. Optimized multimodal neurological image fusion based on low-rank texture prior decomposition and super-pixel segmentation. IEEE Trans Instrum Meas. 2022;71:1–9. doi: 10.1109/tim.2022.3165263

[31] Bhardwaj J, Nayak A. Medical Image Fusion Using Lifting Wavelet and Fractional Bird Swarm Optimization. Advances in Intelligent Systems and Computing. Springer Singapore. 2021. p. 277–90. doi: 10.1007/978-981-16-2123-9_21

[32] Mergin AA, Premi MSG. Convolutional neural networks (CNN) with quantum-behaved particle swarm optimization (qpso)-based medical image fusion. Int J Image Grap. 2022;24(05). doi: 10.1142/s0219467823400053

[33] Kaur M, Singh D. Multi-modality medical image fusion technique using multi-objective differential evolution based deep neural networks. J Ambient Intell Humaniz Comput. 2021;12(2):2483–93. doi: 10.1007/s12652-020-02386-0 32837596 PMC7414903

[34] SJ P, Prakash HN. A Features fusion approach for neonatal and pediatrics brain tumor image analysis using genetic and deep learning techniques. Int J Onl Eng. 2021;17(11):124–40. doi: 10.3991/ijoe.v17i11.25193

[35] Das M, Gupta D, Radeva P, Bakde AM. Multimodal image sensor fusion in a cascaded framework using optimized dual channel pulse coupled neural network. J Ambient Intell Human Comput. 2022;14(9):11985–2004. doi: 10.1007/s12652-022-03749-5

[36] Deb K. Multi-objective optimisation using evolutionary algorithms: an introduction. Multi-objective Evolutionary Optimisation for Product Design and Manufacturing. Springer London. 2011. p. 3–34. doi: 10.1007/978-0-85729-652-8_1

[37] J. C. Tillet, T. M. Rao, F. Sahin, and RRao M. “Darwinian particle swarm optimization”. Indian Int Conference on Art Intelligence. 2005. pp. 1474–87.

[38] Mahima NB, Padmavathi MV, Karki. “Feature extraction using DPSO for medical image fusion based on NSCT,” *2017 2nd IEEE International Conference on Recent Trends in Electronics, Information & Communication Technology (RTEICT)*. Bangalore, India; 2017. pp. 265–9.

[39] Sabatier J, Agrawal OP, Machado JAT. Advances in Fractional Calculus. Springer Netherlands. 2007. doi: 10.1007/978-1-4020-6042-7

[40] Couceiro MS, Rocha RP, Ferreira NMF, Machado JAT. Introducing the fractional-order Darwinian PSO. SIViP. 2012;6(3):343–50. doi: 10.1007/s11760-012-0316-2

[41] Solteiro Pires EJ, Tenreiro Machado JA, de Moura Oliveira PB, Boaventura Cunha J, Mendes L. Particle swarm optimization with fractional-order velocity. Nonlinear Dyn. 2010;61(1–2):295–301. doi: 10.1007/s11071-009-9649-y

[42] Huang J, Shu Q, Zhu X, Shi X, Zhou L, Liu H. A fast frequency domain approximation method for variable order fractional calculus operator based on polynomial fitting. In: 2018 37th Chinese Control Conference (CCC). 2018; 10180–5. doi: 10.23919/chicc.2018.8483651

[43] Puchalski B. Neural Approximators for Variable-Order Fractional Calculus Operators (VO-FC). IEEE Access. 2022;10:7989–8004. doi: 10.1109/access.2022.3143893

[44] Li X, Song S, Zhang H. Evolutionary multiobjective optimization with clustering-based self-adaptive mating restriction strategy. Soft Comput. 2018;23(10):3303–25. doi: 10.1007/s00500-017-2990-z

[45] Li J, Guo X, Lu G, Zhang B, Xu Y, Wu F, et al. DRPL: Deep Regression Pair Learning For Multi-Focus Image Fusion. IEEE Trans Image Process. 2020;10.1109/TIP.2020.2976190. doi: 10.1109/TIP.2020.2976190 32142440

[46] Singh S, Anand RS. Multimodal Medical Image Fusion Using Hybrid Layer Decomposition With CNN-Based Feature Mapping and Structural Clustering. IEEE Trans Instrum Meas. 2020;69(6):3855–65. doi: 10.1109/tim.2019.2933341

[47] Hermessi H, Mourali O, Zagrouba E. Convolutional neural network-based multimodal image fusion via similarity learning in the shearlet domain. Neural Comput & Applic. 2018;30(7):2029–45. doi: 10.1007/s00521-018-3441-1

